# Complete mitochondrial genome of *Oxyartes lamellatus* (Phasmida: Lonchodidae: Necrosciinae)

**DOI:** 10.1128/mra.01041-25

**Published:** 2026-02-27

**Authors:** Shan Li, Yanting Qin, Ting Luo, Xun Bian

**Affiliations:** 1 Ministry of Education, Key Laboratory of Rare and Endangered Species and Environmental Protection, Guangxi Normal University 12388, Guilin, China; University of California Riverside, Riverside, California, USA

**Keywords:** mitogenome, *Oxyartes*, Lonchodidae, Longzhou

## Abstract

The length of the newly sequenced *Oxyartes lamellatus* was 16,291 bp, and it contained 13 protein-coding genes (PCGs), 2 ribosomal genes (rRNAs) and 22 transfer RNA genes (tRNAs). The AT content was rich, accounting for 79.9% of the total length.

## ANNOUNCEMENT

The species *Oxyartes lamellatus* Kirby, 1904 (1) is a kind of relatively robust-bodied stick insects, and it belongs to *Oxyartes* Stål, 1875 (2), Necrosciinae, Lonchodidae, Phasmida. So far, the species and even its genus have mainly been focused on morphological description, without the complete mitogenome being reported, so this study sequenced and annotated the complete mitochondrial genome of *O. lamellatus*.

We collected one female individual of *Oxyartes* in Longzhou County (22.523,697°N, 106.845,627°E, 218 m) and identified it as *O. lamellatus* based on the characteristics of its fifth abdominal tergum with posterior humps, pronotum with spines, and tiny and indistinct forewing (3). We preserved it in 100% ethanol at −4° in the laboratory of Guangxi Normal University, and the DNA extraction was performed using muscles of thoracic and hind leg with the TIANamp Genomic DNA Kit (TIANGEN). Valid DNA samples were selected and sent to Berry Genomics (Beijing, China), where high-throughput sequencing was performed on the Illumina HiSeq 2500 platform with a 150 bp paired-end (PE) read length. This generated a total of 7.7 million (M) PE reads per sample, with an average coverage depth of approximately 141,830×. For quality control, the adapters were trimmed, and the reads with phred quality <Q5 and those with *N* base number >3 were filtered out using fastq v.0.20.0 (4). Company-returned data were standardized in CLC Genomics Workbench 12 (5) for seamless downstream integration. Compared with the 61 available mitochondrial genomes of Gryllacrididae deposited in NCBI, the sequence with the highest similarity to the newly assembled mitochondrial genome was selected as a reference. Based on the reference sequence, the newly sequenced genome was assembled into a circular mitochondrial DNA using NOVOplasty 4.2.1 (6). Then, the data assembled into a circular form were annotated using MITOS2 (7) from the Galaxy platform with the genetic code of the fifth invertebrate and RefSeq 89 Metazoa. The annotated data were proofread using MEGA V.11.0 to ensure the correctness of protein translation, yielding standardized mitochondrial genome data for *O. lamellatus* (8).

**Fig 1:**
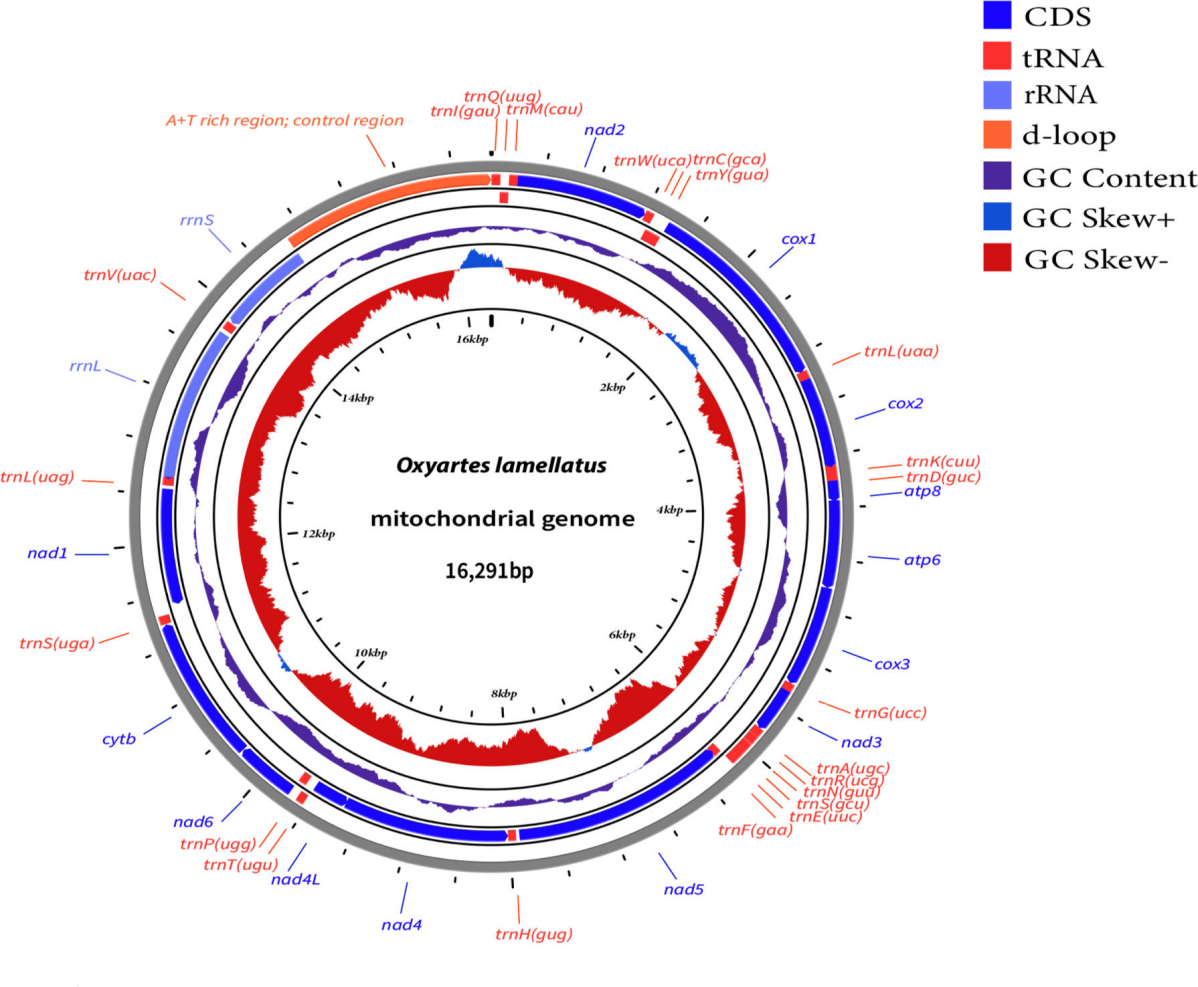
The genome map of *O. lamellatus* was drawn using Proksee (https://proksee.ca/) (14).

The complete mitochondrial genome of the sequenced sample was 16,291 bp in length, containing 13 protein-coding genes (PCGs), 2 ribosomal gene (rRNA) genes, and 22 transfer RNA (tRNA) genes. (Fig. 1) All 13 protein-coding genes used ATN (ATA, ATT, and ATG) as start codons. As for stop codons, *nad3* used TAG; *cox1* and *cox2* used T (as the initial termination signal that can form a complete termination codon via polyadenylation) (9, 10), similarly to many insects (11); the rest used TAA. The AT content was high, reaching 79.9%, while the GC content was only 20.1% (Table 1), similarly to other stick insects (12, 13). The codon with the highest RSCU value was UUA, indicating its strongest preference in synonymous codon usage.

**TABLE 1:** The lengths and base composition ratios of each part in the mitochondrial genome of *O. lamellatus*

	Length (bp)	A%	T%	C%	G%	AT%	GC%	GT%	AT skew	GC skew
Full genome	16,291	45.1	34.8	11.9	8.2	79.9	20.1	43	0.129	−0.179
PCGs	11,103	35.8	43.4	10.3	10.5	79.2	20.8	53.9	−0.095	0.013
rRNAs	2,044	33.9	46.2	7	12.9	80.1	19.9	59.1	−0.155	0.297
tRNAs	1,474	41.5	39.1	8.1	11.3	80.6	19.4	50.4	0.029	0.161

## Data Availability

The complete mitochondrial genome of *O. lamellatus* is available in GenBank under accession number PX309546, and the biosample number, bioproject number, SRA number are SAMN51172106, PRJNA1320832, and SRR35276853, respectively.
